# Insulin secretion: The nitric oxide controversy

**DOI:** 10.17179/excli2020-2711

**Published:** 2020-09-08

**Authors:** Sevda Gheibi, Asghar Ghasemi

**Affiliations:** 1 Department of Clinical Sciences in Malmö, Unit of Molecular Metabolism, Lund University Diabetes Centre, Clinical Research Center, Malmö University Hospital, Lund University, Malmö, Sweden; 2 Endocrine Physiology Research Center, Research Institute for Endocrine Sciences, Shahid Beheshti University of Medical Sciences, Tehran, Iran

**Keywords:** nitric oxide, insulin secretion, insulin synthesis

## Abstract

Nitric oxide (NO) is a gas that serves as a ubiquitous signaling molecule participating in physiological activities of various organ systems. Nitric oxide is produced in the endocrine pancreas and contributes to synthesis and secretion of insulin. The potential role of NO in insulin secretion is disputable - both stimulatory and inhibitory effects have been reported. Available data indicate that effects of NO critically depend on its concentration. Different isoforms of NO synthase (NOS) control this and have the potential to decrease or increase insulin secretion. In this review, the role of NO in insulin secretion as well as the possible reasons for discrepant findings are discussed. A better understanding of the role of NO system in the regulation of insulin secretion may facilitate the development of new therapeutic strategies in the management of diabetes.

## Introduction

Nitric oxide (NO) is a short-lived, volatile gas that also serves as a ubiquitous signaling molecule involved in a variety of biological functions, which, if altered, could contribute to the genesis of many pathological conditions (Lundberg et al., 2018[77]). One area of recent interest is the potential role of NO in the regulation of insulin synthesis and secretion; these effects are, however, highly complex as both inhibitory and stimulatory effects of NO on insulin secretion have been reported (Nystrom et al., 2012[95]; Sansbury and Hill, 2014[109]; Bahadoran et al., 2020[10]). 

Nitric oxide through increasing intracellular Ca^2+^ levels or via *S*-nitrosylation of glucokinase and syntaxin 4, as well as vasodilation of islet vasculature, increases insulin secretion (Laffranchi et al., 1995[67]; Rizzo and Piston, 2003[105]; Wiseman et al., 2011[134]; Nystrom et al., 2012[95]; Kruszelnicka, 2014[65]). Decreased NO bioavailability has been shown in obesity and type 2 diabetes in both animal and human studies (Wu and Meininger, 2009[136]; Jiang et al., 2014[59]; Sansbury and Hill, 2014[109]; Bakhtiarzadeh et al., 2018[12]) and restoration of NO levels has many favorable metabolic effects in type 2 diabetes (Carlstrom et al., 2010[18]; Gheibi et al., 2017[40], 2018[41], 2019[42]). These studies suggest that NO through modulation of insulin secretion and its signaling pathways may be a potential target for the treatment of type 2 diabetes. By contrast, inhibition of islet NO synthase (NOS) activity is accompanied by an increase in glucose-stimulated insulin secretion (GSIS) (Panagiotidis et al., 1995[100]; Akesson et al., 1999[1]; Henningsson et al., 2000[49], 2002[52]; Eckersten and Henningsson, 2012[28]). Moreover, GSIS has been shown to be suppressed by different concentrations of NO donors (Panagiotidis et al., 1995[100]; Antoine et al., 1996[5]; Akesson and Lundquist, 1999[2]), indicating a negative role of NO in insulin secretion. 

The controversy of NO's role in insulin secretion may depend on the use of various β-cell lines with different qualitative/quantitative secretory reaction patterns, incubation of islets/β-cells in high or low glucose media, using different NOS inhibitors, or different types of extracellular/intracellular NO donors. Also varying enzymatic activities of the different isoforms of NOS may be of importance. This review focuses on the role of NO in the regulation of insulin secretion as well as the possible factors and reasons which may contribute to discrepancies in the results between different studies.

## An Overview of NO Production and Function

### Production of NO

Nitric oxide is produced in NOS-dependent and independent pathways (Aronstam et al., 1995[6]; Ghasemi and Jeddi, 2017[39]). In NOS-dependent pathways, NO is produced by three isoforms of NOS, referred to as neuronal (nNOS/NOS1), inducible (iNOS/NOS2), and endothelial (eNOS/NOS3) (Knowles and Moncada, 1994[64]; Cannon, 1998[17]; Yoon et al., 2000[138]; Sansbury and Hill, 2014[109]). L-arginine is the substrate for all isoforms. For NO generation, NOS first hydroxylates L-arginine to N(omega)-hydroxy-L-arginine (L-NOHA) and then oxidizes L-NOHA to L-citrulline and NO (Stuehr, 2004[122]). 

Both nNOS and eNOS are constitutively expressed proteins, collectively termed as cNOS; nNOS is expressed in specific neurons of the brain but also found in skeletal muscle and epithelial cells (Frandsen et al., 1996[34]; McConell et al., 2007[85]). eNOS, which produces relatively low quantities of NO, is expressed at the highest relative abundance in the vascular endothelium (McNaughton et al., 2002[88]; Tanaka et al., 2003[125]). Although iNOS expression is primarily identified in macrophages, it can be induced in virtually any cells or tissues by inflammatory cytokines (Lee et al., 2003[72]; Luiking et al., 2010[76]). A mitochondria-localized NOS isoform has also been reported (Finocchietto et al., 2009[32]), however, its specific contribution remains unclear. Activity of cNOS is regulated by Ca^2+^ and calmodulin; by elevation in intracellular Ca^2+ ^levels, eNOS activity is markedly increased and results in production of NO in a pulsatile manner (Forstermann and Sessa, 2012[33]). Activity of iNOS is not regulated by Ca^2+^ with calmodulin and the enzyme active even at extremely low intracellular Ca^2+^ levels (Forstermann and Sessa, 2012[33]; Berridge, 2014[13]). Once expressed, iNOS produces a large amount of NO. Higher concentrations of NO, produced by iNOS or exogenous NO leads to inhibition of cNOS (Schwartz et al., 1997[111]; Sansbury and Hill, 2014[109]), as lower concentrations of NO are required for inhibition of cNOS than for iNOS inhibition (Schwartz et al., 1997[111]).

In NOS-independent pathways, NO is produced from nitrate and nitrite; oxidation of endogenous NO and diet are two major sources of nitrate in mammals (Lundberg and Weitzberg, 2013[78]). About 25 % of circulating nitrate is actively taken up by the salivary glands and then reduced to nitrite by the oral commensal bacteria (Lundberg et al., 2008[80]). After oral loading, nitrate/nitrite is rapidly absorbed in the duodenum and jejunum (Carlstrom et al., 2010[18]; Kevil et al., 2011[63]). In the stomach, part of the nitrite is reduced to NO but most of it is absorbed into the circulation (Weitzberg and Lundberg, 1998[131]; Dauncey, 2012[25]). Nitrite reduction to NO in blood and tissues could be enzymatic or non-enzymatic and is generally enhanced during hypoxic, ischemic, and acidic conditions (Lundberg et al., 2008[80]; Lundberg and Weitzberg, 2010[79]).

### Nitric oxide signaling pathways

Actions of NO can be cyclic guanosine monophosphate (cGMP)-dependent and cGMP-independent (mostly reactive nitrogen species-mediated) (Cordes et al., 2009[21]). The cGMP-dependent signaling pathway is the most important physiologic signaling pathway activated by NO (Pacher et al., 2007[97]; Omar et al., 2016[96]). In this pathway, only low concentrations of NO (5-10 nM) are required to activate guanylyl cyclase (GC) (Murad, 2006[91]; Pacher et al., 2007[97]), which converts guanosine triphosphate to cGMP (Murad, 2006[91]). Guanylyl cyclase has two isoforms: soluble (sGC is cytosolic) and membrane (particulate), of which, sGC is the receptor for NO (Murad, 2006[91]). Binding of two NO molecules is necessary for full activation of sGC; the first molecule binds to the β-subunit of sGC at picomolar affinity, partially activating it to ~15 % of its maximal activity (Horst and Marletta, 2018[54]); full activation of sGC depends on the NO concentration and occurs following binding of a second molecule of NO, which happens at nanomolar affinity; a conformational change in sGC following NO binding is the rate-limiting step for its activation; switching between partially and fully activated states is responsible for rapid activation and deactivation of sGC (Bahadoran et al., 2020[9]). The second site of sGC releases NO when cellular NO concentrations fall with the enzyme returning to its partially active state (Horst and Marletta, 2018[54]). Elevated cGMP levels activate the downstream elements of the NO signaling pathway (PKG, cGMP-gated cation channels and cGMP-regulated phosphodiesterases) and mediate its physiological actions (Gheibi et al., 2018[43], 2020[44]).

In addition to the NO/cGMP/PKG signaling pathway, nitrosative post-translational modifications such as *S*-nitrosylation, which is a reversible covalent attachment of NO to the cysteine residues of proteins, is a key mechanism for NO signaling (Crawford and Guo, 2005[22]; Berridge, 2014[13]). *S*-nitrosylation activates or inhibits protein function and therefore can be beneficial or detrimental (Wiseman and Thurmond, 2012[135]; Zheng et al., 2016[139]). Despite the presence of cysteine residues on almost all proteins and production of NO by most cells, only some proteins are nitrosylated (Mannick and Schonhoff, 2002[82]). The specificity of *S*-nitrosylation depends on the presence of metal ions (Mg^2+^ or Ca^2+^), local pH, and acid-base motifs (Altaany et al., 2014[4]).

## Overview of Insulin Synthesis and Secretion

Insulin is produced by β-cells in the islets of Langerhans; each islet contains ~1000 β-cells and each β-cell contains ~10,000-13,000 granules with a single granule containing 8 fg (10^-18^ mol or 10^6^ molecules) of insulin (Renström and Rorsman, 2007[104]; Eliasson et al., 2008[31]). The insulin gene encodes a single-chain precursor known as preproinsulin containing the signal peptide, insulin A-chain, C-peptide, and insulin B-chain (Fu et al., 2013[35]). The signal peptide is located at the N-terminus of preproinsulin and interacts with cytosolic ribonucleoprotein signal recognition particles (SRP), which facilitate preproinsulin translocation across the rough endoplasmic reticulum (RER) membrane into its lumen (Fu et al., 2013[35]). In the RER, the signal peptide is cleaved by a signal peptidase to yield proinsulin; proinsulin is then transported to the Golgi apparatus and packaged into the secretory granules where the conversion of proinsulin to native insulin and C-peptide begins before they are stored in the mature secretory granules (Fu et al., 2013[35]). 

Only 1-3 % of secreted insulin from the islets of Langerhans is in the form of proinsulin but due to its long half-life, it accounts for 5-30 % of the insulin-like molecules circulating in the blood. Additionally, because of similarities in the structure of proinsulin and insulin, proinsulin can bind with some affinity to the insulin receptor, producing up to 5-10 % of the metabolic activity induced by insulin (Weiss et al., 2000[130]). 

An elevated blood glucose level is the main stimulus for insulin secretion from β-cells. Glucose enters the pancreatic β‐cells via a low affinity glucose transporter (GLUT-2 in rodents and GLUT-1 as well as GLUT-3 in human) (McCulloch et al., 2011[86]); glucose is phosphorylated by glucokinase, and pyruvate is generated through glycolysis in the cytoplasm (Fu et al., 2013[35]). Pyruvate is then metabolized by pyruvate carboxylase and pyruvate dehydrogenase in the mitochondria, ultimately leading to an increase in the cytoplasmic adenosine triphosphate (ATP)/adenosine diphosphate (ADP) ratio, which closes ATP‐sensitive K^+^ channels (K_ATP_ channels) (Eliasson et al., 2008[31]). In β‐cells, K_ATP_ channels are the primary determinants of the membrane potential; closure of these channels causes membrane depolarization and the subsequent activation of L‐type voltage‐dependent Ca^2+^ channels (VDCC). This results in elevation of the cytosolic free Ca^2+^ concentration ([Ca^2+^]_i_), followed by insulin granule content release into the circulation (Fu et al., 2013[35]).

Insulin granule content release, or exocytosis, into the circulation includes several steps. Firstly, granule translocation to the plasma membrane followed by tethering, docking, priming, and fusion of the granules (Gerber and Sudhof, 2002[38]; Martin-Urdiroz et al., 2016[83]). Insulin secretion follows a characteristic biphasic time course: (1) the first phase involves the plasma-membrane fusion of vesicles that are primed at the vicinity of the plasma membrane, termed the readily releasable pool (RRP); (2) the second phase involves the mobilization of intracellular granules that are deeper within the cell from the plasma membrane (Wang and Thurmond, 2009[129]).

### Insulin granule exocytosis 

#### Translocation to the plasma membrane

Motor proteins transport insulin-containing vesicles toward the membrane (Martin-Urdiroz et al., 2016[83]). This movement is both random and directed along microtubules (Renström and Rorsman, 2007[104]), which are distributed throughout the cell, except in the periphery, where there is an actin web (Renström, 2011[103]). Anterograde (forward) movement of granules depends on kinesin-1 with a maximum velocity of 600-800 nm/sec (Renström and Rorsman, 2007[104]); kinesin-3 may provide a very fast (1000 nm/sec) insulin granule translocation to the plasma membrane (Renström and Rorsman, 2007[104]). As kinesins are ATP-dependent, glucose, by increasing the cytosolic ATP level, increases kinesin activity and therefore insulin secretion (Wang and Thurmond, 2009[129]). Movement of insulin granules in the reverse direction is achieved through dynein (Renström and Rorsman, 2007[104]). Under basal conditions, actin filaments under the plasma membrane prevent insulin hypersecretion (Renström and Rorsman, 2007[104]). However, glucose transiently causes F-actin remodeling, regulated by the small Rho-family GTPases, Cdc42 and Rac1. Cdc42 is activated within 3 minutes of glucose stimulation while Rac1 activation is not apparent until 15-20 minutes after stimulation (Wang and Thurmond, 2009[129]). 

#### SNARE-mediated exocytosis

Insulin vesicle exocytosis is mediated by soluble *N*-ethylmaleimide-sensitive factor attachment protein receptor (SNARE) proteins (Gaisano, 2017[37]), which can be divided into two categories: vesicle (v)-SNAREs from the vesicle membrane, and target (t)-SNAREs from the target membrane. t-SNARE, includes the syntaxin and synaptosome associated protein-25 (SNAP-25) families, while v-SNAREs refers to vesicle-associated membrane proteins (VAMPs) (Jewell et al., 2010[58]). 

Tethering is the initial contact between a vesicle and the plasma membrane and is mediated by the exocyst complex (Liu and Guo, 2012[74]; Martin-Urdiroz et al., 2016[83]), which is a conserved octameric complex that tethers exocytic vesicles to the plasma membrane prior to fusion. This complex contains Sec (for secretion) and Exo (for exocyst related) proteins (Liu and Guo, 2012[74]; Xie et al., 2013[137]; Martin-Urdiroz et al., 2016[83]). In docking and attachment to the plasma membrane, Munc18 (mammalian uncoordinated-18) binds to syntaxin and recruits SNAP-25 to the plasma membrane (Shibasaki et al., 2014[114]). Rab3A, a small GTPase, with its interacting partner (Rim2α) binds to Munc18 and plays an important role in docking (Shibasaki et al., 2014[114]). v-SNARE VAMP-2 (also known as synaptobrevin) pairs with the t-SNAREs syntaxin and SNAP-23/25 to form the SNARE core complex. Prior to formation of the SNARE complex, the accessory protein Sec1/Munc18 provides a bridge between v-SNARE and t-SNARE so that the space between membranes is closed (Martin-Urdiroz et al., 2016[83]). Munc18 proteins are essential regulators of SNARE-mediated exocytosis and reduction in Munc18/SNARE complex has been implicated in the loss of first phase of insulin secretion in type 2 diabetes (Gaisano, 2014[36]). Priming, in which granules become ready for the fusion process, is an ATP-dependent reaction necessary for exocytosis (Renström, 2011[103]; Shibasaki et al., 2014[114]). In priming, SNARE proteins form a complex in preparation for Ca^2+^-triggered fusion (Gaisano, 2014[36]). Following Ca^2+^ entry, synaptotagmins act as Ca^2+^ sensors (Sollner, 2003[117]) and also promote fusion pore formation (Gaisano, 2014[36]) leading to insulin secretion. The primary Ca^2+^ sensor in β-cells is synaptotagmin-7 (Roder et al., 2016[106]).

## Nitric Oxide and the Endocrine Pancreas

### Nitric oxide production in endocrine pancreas

Pancreatic β-cells express all three isoforms of NOS (Table 1(Tab. 1); References in Table 1: Akesson et al., 1999[1]; Bachar et al., 2010[8]; Corbett and McDaniel, 1995[20]; Henningsson et al., 2002[52]; Hogan et al., 2016[53]; Hu et al., 2017[55]; Jimenez-Feltstrom et al., 2005[60]; Lajoix et al., 2002[68]; Mezghenna et al., 2011[90]; Nakada et al., 2003[92]; Spinas et al., 1998[119]; Tsuura et al., 1998[127]) (Shimabukuro et al., 1997[115]; Lajoix et al., 2001[68]; Henningsson et al., 2002[52]; Nakada et al., 2003[92]; Novelli et al., 2004[93]; Jimenez-Feltstrom et al., 2005[60]; Kurohane Kaneko and Ishikawa, 2013[66]; Lundberg and Weitzberg, 2013[78]; Broniowska et al., 2014[15]). nNOS is usually considered to be the major isoform with multifunctional properties in the pancreas; it is mainly found in insulin secretory granules, but also in the mitochondrion and the nucleus (Spinas et al., 1998[119]; Lajoix et al., 2001[68]; Mezghenna et al., 2011[90]). The activity of nNOS is regulated positively by Ca^2+^/calmodulin, glucose, and palmitate (Salehi et al., 1996[107]; Bachar et al., 2010[8]) and negatively by cytokines (Gurgul-Convey et al., 2012[46]). There are few studies focusing on eNOS function in β-cells (Kurohane Kaneko and Ishikawa, 2013[66]). The presence of this NOS isoform has however been reported in rodent β-cells (Nakada et al., 2003[92]; Hogan et al., 2016[53]). At basal glucose concentrations, iNOS is not detectable in β-cells but its expression is increased following exposure to higher cytoplasmic glucose concentrations (Henningsson et al., 2002[52]; Jimenez-Feltstrom et al., 2005[60]).

At a normoglycemic concentration of glucose (7 mM), a small amount of NO is produced, which is derived from cNOS (Henningsson et al., 2002[52]); iNOS activity has been shown to be increased at >10 mM glucose, which is in strong agreement with its protein expression (Henningsson et al., 2002[52]). In addition to the effects of glucose concentration, the potential role of fasting on NO production has also been reported (Eckersten and Henningsson, 2012[28]). Indeed, in islets from freely fed mice, iNOS activity is very low (1.1 ± 1.1 pmol NO/min/mg protein) but increases following fasting (15 ± 1.3 pmol NO/min/mg protein) both at low and high glucose concentrations (Henningsson et al., 2000[49]; Eckersten and Henningsson, 2012[28]). There was no influence of starvation on islet cNOS activity (19.3±1.4 pmol citrulline×mg protein^−1^×min^−1^) at basal glucose concentration (7 mM) but increased at 20 mM glucose (30.7±3.4 pmol citrulline×mg protein^−1^×min^−1^) (Eckersten and Henningsson, 2012[28]).

### Nitric oxide and insulin secretion

The potential role of NO in insulin secretion has been widely disputed, and the results obtained are highly controversial. It has been reported that NO stimulates (Laychock et al., 1991[70]; Schmidt et al., 1992[110]; Willmott et al., 1995[133]; Ding and Rana, 1998[26]; Spinas et al., 1998[119]; Matsuura et al., 1999[84]; Spinas, 1999[118]; Smukler et al., 2002[116]; Nystrom et al., 2012[95]; Gheibi et al., 2017[40]), inhibits (Panagiotidis et al., 1992[101], 1994[99], 1995[100]; Gross et al., 1995[45]; Akesson and Lundquist, 1999[2]; Akesson et al., 1996[3], 1999[1]; Antoine et al., 1996[5]; Salehi et al., 1996[107], 1998[108]; Henningsson and Lundquist, 1998[51]; Henningsson et al., 1999[48], 2000[49], 2001[50]; Tsuura et al., 1998[127]) or has negligible effect (Jones et al., 1992[61]; Gheibi et al., 2018[41]) on insulin secretion in studies using islets and β-cell lines with various types and concentrations of NOS inhibitors and NO donors (Table 2(Tab. 2); References in Table 2: Akesson et al., 1999[1]; Ding and Rana, 1998[26]; Gheibi et al., 2017[40], 2018[41]; Henningsson et al., 2002[52]; Jiminez-Feltstrom et al., 2005[60]; Laychock et al., 1991[70]; Mezghenna et al., 2011[90]; Nystrom et al., 2012[95]; Panagiotidis et al., 1995[100]; Salehi et al., 1998[108]; Smukler et al, 2002[116]; Spinas et al., 1998[119]; Tsuura et al., 1998[127]). In the following sections, we will discuss in more detail how NO may stimulate or inhibit insulin secretion.

#### Stimulatory effect of NO on insulin secretion

The initial evidence that NO played a role in the regulation of insulin secretion came from Laychock and colleagues in 1991 (Laychock et al., 1991[70]). They found that sodium nitroprusside by increasing the cGMP level in rat islets, stimulated insulin secretion, while inhibition of NOS decreased glucose- and arginine-induced cGMP release (Laychock et al., 1991[70]). This was further supported by the finding that L-arginine-derived NO increases basal and GSIS in isolated mouse islets (Henningsson and Lundquist, 1998[51]; Henningsson et al., 1999[48]) and the glucose-responsive clonal pancreatic β-cell line HIT-T15 (Schmidt et al., 1992[110]). A concomitant release of insulin and NO is induced by L-arginine in the presence of D-glucose, with the median effective arginine concentrations (EC_50_) for insulin and NO release equal to 150 µM and 50 µM, respectively, both of which are within the physiological range of circulating L-arginine levels. Interestingly, L-arginine also decreases the EC_50_ for D-glucose's stimulation of both NO and insulin release (from 15 mM to 5 mM) (Schmidt et al., 1992[110]).

**Figure 1:**
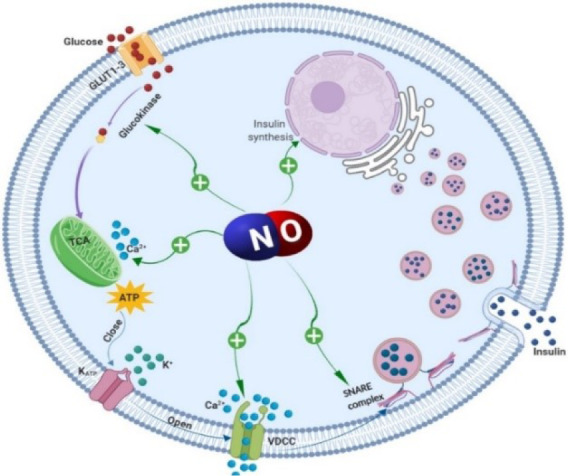
Stimulatory effects of nitric oxide (NO) on insulin secretion. Glucose enters the pancreatic β‐cells via glucose transporter type 2 (GLUT-2) and is phosphorylated by glucokinase. Pyruvate is generated through glycolysis and is subsequently further metabolized in the mitochondria, which increases cytoplasmic adenosine triphosphate (ATP) level. Increased ATP level closes ATP‐sensitive K^+^ channels (K_ATP_ channels). Closure of these channels causes membrane depolarization and the subsequent activation of L‐type voltage‐dependent Ca^2+^ channels (VDCC); elevation of cytosolic free Ca^2+^ concentration is followed by the release of insulin granules into the circulation. Nitric oxide through increasing intracellular Ca^2+^ levels (activation of VDCC and release from the mitochondria), insulin synthesis, or via S-nitrosylation of glucokinase and soluble N-ethylmaleimide-sensitive factor attachment protein receptor (SNARE) protein syntaxin 4 increases insulin secretion.

Endogenously produced NO also plays an important role in insulin secretion. Indeed, scavenging of NO with cPTIO (carboxy-2-phenyl-4, 4, 5, 5-tetramethylimidazoline-1-oxyl 3-oxide) in highly glucose-responsive INS-1 cells, is able to significantly reduce the stimulation provided by 15 mM glucose (by ~40 %) (Smukler et al., 2002[116]), highlighting the involvement of endogenously produced NO in secretagogue-induced insulin secretion under physiological conditions. It also seems that the early phase of insulin secretion is NO-dependent as scavenging of endogenous NO or inhibition of NOS with L-NMMA (NG-Monomethyl-L-arginine, monoacetate) in rat pancreatic islets blunts the early insulin peak by 60-65 % and 46 %, respectively (Spinas et al., 1998[119]). This finding may also explain why some studies looking into accumulated insulin release in pancreatic islets argue against a stimulatory effect of NO on insulin release. The mechanism by which NO stimulates insulin secretion is shown in Figure 1(Fig. 1) and also discussed below.

#### Nitric oxide increases insulin synthesis

Increased insulin synthesis has been reported following NO treatment both in Min6 β-cells as well as in intact pancreatic islets (Campbell et al., 2007[16]). High fat diet STZ-induced diabetic rats were shown to have low islet insulin content; long term nitrite supplementation in these rats increased islet insulin content, indicating increased insulin synthesis (Gheibi et al., 2017[40]). Nitric oxide (NO gas) stimulates the activity of the insulin gene promoter in Min6 β-cells with a maximal 2.5-fold stimulation at 24 h, an effect which is reversed using PI3-kinase inhibitor (wortmannin), indicating that PI3-kinase activity is essential for the effects of NO on insulin gene promoter activity (Campbell et al., 2007[16]). In addition, NO (NO gas) increases endogenous insulin gene expression in Min6 cells and isolated rat islets of Langerhans, promoting the nuclear accumulation of PDX-1 (pancreatic and duodenal homeobox factor-1) and its subsequent binding to the insulin gene promoter (Campbell et al., 2007[16]). PDX-1 plays an important role in β-cells by linking glucose metabolism to events in the β-cell nucleus; in response to elevated glucose levels, PDX-1 is mobilized from its resting position in the cytoplasm into the β-cell nucleus, where it binds and activates the insulin gene promoter (Macfarlane et al., 1999[81]). By contrast, treatment with *S*-nitrosoglutathione (GSNO), a source of bioavailable NO, has no effect on islet insulin content in human and rat islets (Hadjivassiliou et al., 1998[47]). Similarly, L-NAME (N(ω)-nitro-L-arginine methyl ester) does not affect the attenuation of proinsulin synthesis, or the depletion of islet insulin content induced by palmitate (Bachar et al., 2010[8]).

#### Nitric oxide increases cGMP levels

Increased cGMP level is a putative mechanism through which NO exerts its action; L-arginine with D-glucose, in the absence or presence of a non-selective phosphodiesterase inhibitor (isobutyl-methylxanthine), increase the level of cGMP in rat pancreatic islets and in HIT-T15 cells (Schmidt et al., 1992[110]). In addition, direct exposure to the NO donor, 3-morpholinosydnonimine (SIN-1), also elevates basal cGMP levels in HIT-T15 cells (Schmidt et al., 1992[110]). The stimulatory effect of hydroxylamine on insulin secretion is abolished by GC inhibition with ODQ (1H-[1, 2, 4]oxadiazolo[4, 3-a]quinoxalin-1-one) while that stimulatory ability of NO is mimicked by the activation of GC using YC-1 (3-(5-hydroxymethyl-3-furyl)-1-benzylindazole), and by the membrane-permeable cGMP analog (8-(4-chlorophenylthio)-cGMP) in INS-1 cells and rat islets (Smukler et al., 2002[116]), again supporting the notion that NO acts principally by stimulating GC. Elevated cGMP levels increase Ca^2+^ influx at 7 mM but not at 2.8 mM of glucose. This appears to result from activation of VDCC in rat pancreatic β-cells as the [Ca^2+^]_i_ elevation is abolished by nicardipine, a dihydropyridine class of Ca^2+^ channel blockers (Matsuura et al., 1999[84]). Interestingly, it is believed that low NO levels act through the NO/sGC/cGMP pathway while high NO levels exert their effects independently of cGMP (Lazo-de-la-Vega-Monroy and Vilches-Flores, 2014[71]). Moreover, the NO/sGC/cGMP pathway participates in other positive effects for β-cells, such as enhancing islet blood flow (Nystrom et al., 2012[95]) and decreasing apoptosis (Tejedo et al., 2001[126]).

#### Nitric oxide increases intracellular Ca^2+^ levels

Nitric oxide increases intracellular Ca^2+^ levels through mobilization of Ca^2+^ from intracellular stores, such as the endoplasmic reticulum and mitochondria (Laffranchi et al., 1995[67]; Willmott et al., 1995[133]) or through inhibition of K_ATP_ channels and subsequent membrane depolarization, leading to opening of VDCCs (Smukler et al., 2002[116]). Application of diazoxide, a specific activator of K_ATP_ channels, is able to inhibit hydroxylamine-stimulated insulin release in INS-1 cells (Smukler et al., 2002[116]); in this study, Ca^2+^ imaging with Fura-2 demonstrated that hydroxylamine stimulates a spiking, oscillatory elevation of [Ca^2+^]_i_, similar to that seen with glucose (15 mM) and this [Ca^2+^]_i_ response occurs simultaneously with membrane depolarization (Smukler et al., 2002[116]).

Nitric oxide (NO gas), by binding to cytochrome *c *and/or cytochrome oxidase inhibits the mitochondrial respiratory chain, causing decreased mitochondrial membrane potential and mobilization of Ca^2+^ from mitochondria (Schweizer and Richter, 1994[112]). Addition of low concentrations of NO to INS-1 cells results in a rapid increase in insulin secretion, which is paralleled by decreased mitochondrial membrane potential and also an intermittent rise of cytosolic Ca^2+^. Furthermore, NO-induced Ca^2+^ release from the mitochondria and increased insulin secretion are independent of extracellular Ca^2+^, as chelation of intracellular but not of extracellular Ca^2+^, decreases NO-induced insulin secretion (Laffranchi et al., 1995[67]). In addition, when intracellular Ca^2+^ levels are raised in advance, the NO-induced cGMP elevation restores normal intracellular Ca^2+^ levels via Ca^2+^ sequestration into the endoplasmic reticulum (Matsuura et al., 1999[84]), suggesting that NO, via elevation of cGMP, not only increases insulin secretion (Ishikawa et al., 2003[56]), but also protects β-cells from excessive Ca^2+^ increases, which may lead to apoptosis (Kaneko et al., 2003[62]). 

Elevated Ca^2+^ levels also increase NO production in INS-1 cells (Smukler et al., 2002[116]) and it is interesting to note that induction of NO production is an early event in the onset of the insulin secretion process. Although NO production can be detected prior to an increase in intracellular Ca^2+^ levels (Nunemaker et al., 2007[94]), it is not clear that this happens because the Ca^2+^ levels required for NOS activation are actually below the threshold for detection by Fura-2. Indeed, this interpretation is compatible with a body of literature which demonstrates requirement of Ca^2+^/calmodulin binding for the activation of NOS (Spratt et al., 2007[120]). As such, one could speculate that producing NO at such an early point would support the idea of a positive role, rather than a negative one, and that for negative regulation to occur, a certain threshold must be crossed.

#### Nitric oxide acts through S-nitrosylation 

Nitric oxide through* S*-nitrosylation of glucokinase (at cysteine-371) and syntaxin 4 (at cysteine-141) facilitates GSIS (Rizzo and Piston, 2003[105]; Wiseman et al., 2011[134]; Kruszelnicka, 2014[65]; Seckinger et al., 2018[113]). Using quantitative imaging of multicolor fluorescent proteins fused to glucokinase, it has been demonstrated that the dynamic association of glucokinase with secretory granules is modulated by NO (Rizzo and Piston, 2003[105]). Indeed, insulin is found to stimulate NO production leading to *S*-nitrosylation of glucokinase in cultured β-cells (βTC3 cells); moreover, inhibition of NOS disrupts glucokinase association with secretory granules and glucokinase conformation (Rizzo and Piston, 2003[105]). It has been demonstrated that elevated glucose and *S*-nitrosylation, both induce the same high-activity glucokinase conformational state I in βTC3 cells (Seckinger et al., 2018[113]). Ultimately, attachment of a nuclear localization signal sequence to NOS in βTC3 cells drives glucokinase to the nucleus in addition to its normal cytoplasmic and granule targeting (Rizzo and Piston, 2003[105]). These data suggest that the regulation of glucokinase localization and activity in pancreatic β-cells is directly related to NO production and that the association of glucokinase with secretory granules occurs through its interaction with NOS.

The SNARE proteins are post-translationally modified by NO, which in turn impacts vesicle exocytosis; NO-stimulated vesicle exocytosis was found to be mediated by activation of the core complex proteins involved in docking and fusion of the vesicles (Meffert et al., 1996[89]). In experiments using recombinant proteins, NO donors (sodium nitroprusside (SNP), acidified sodium nitrite, S-nitrosoglutathione, S-nitrosocysteine and a saturated solution of NO gas) increase formation of the VAMP/SNAP-25/syntaxin 1a core complex and inhibit the binding of n-sec1 to syntaxin 1a (Meffert et al., 1996[89]); these NO donors lower the EC_50_ of VAMP binding to SNAP-25/syntaxin (Meffert et al., 1996[89]). In addition, *S*-nitrosylation of syntaxin 1a (at cysteine-145) seems to be a molecular switch to disrupt Munc18-1 binding to the closed conformation of syntaxin 1a, thereby facilitating its engagement with the membrane fusion machinery (Palmer et al., 2008[98]). Taken together, these activities are predicted to promote vesicle docking/fusion. 

At present, only the t-SNARE protein syntaxin 4 has been shown to be specifically *S*-nitrosylated in pancreatic β-cells; once *S*-nitrosylated, this event facilitates GSIS (Wiseman et al., 2011[134]; Kruszelnicka, 2014[65]). Interestingly, while syntaxin 1 is a target of *S*-nitrosylation in neuronal cells and tissues (Pongrac et al., 2007[102]; Palmer et al., 2008[98]), it is not modified in response to glucose in the pancreatic β-cells, demonstrating that a similar complement of exocytotic proteins results in differing functional rate kinetics across different tissues. The cellular content of *S*-nitrosylated syntaxin 4 has been shown to peak acutely within 5 min of glucose stimulation in both human islets and MIN6 β-cells (Wiseman et al., 2011[134]). Mutation in the gene encoding cysteine-141-syntaxin 4 prevents *S*-nitrosylation induced by the NO donor (*S*-nitrosoglutathione), fails to exhibit glucose-induced activation and VAMP2 binding, and consequently fails to potentiate insulin release (Wiseman et al., 2011[134]). In addition, monitoring of single-cell exocytosis, using the styryl dye, FM1 43, demonstrates that NO (hydroxylamine, SNP, and SIN-1) exerts a rapid stimulatory effect on insulin secretion from INS-1 cells (Smukler et al., 2002[116]).

#### Nitric oxide increases islet blood flow

Increased islet blood flow, which supplies oxygen and nutrients to the islets, is another mechanism by which NO may increase insulin secretion (Nystrom et al., 2012[95]). Nitric oxide has been suggested as an important regulator of islet blood flow; insulin secretion can be rapidly modulated by changes in rat islet microcirculation (Jansson and Hellerstrom, 1983[57]). The islets have a well-developed vascular network with a highly fenestrated endothelium in capillaries, thus allowing rapid and efficient delivery of oxygen and nutrients to the endocrine cells. Interestingly, islet blood flow can be regulated independently from the exocrine part of the pancreas (Bonner-Weir and Orci, 1982[14]; Jansson and Hellerstrom, 1983[57]). Nystrom and colleagues studied the effects of nitrite on pancreatic rat islet blood flow and dynamic changes in insulin secretion and glycemia (Nystrom et al., 2012[95]). They found that sodium nitrite increases islet blood flow, with no such effect on total pancreatic blood flow; the enhancement of islet blood flow was followed by an increase in plasma insulin concentrations. However, glycemia was unchanged (Nystrom et al., 2012[95]), suggesting mobilization of counterregulatory hormones, such as glucagon and cortisol, opposing insulin action. In addition, nitrite-increased islet blood flow was abolished by a GC inhibitor and a NO scavenger and mimicked by a cGMP agonist (Nystrom et al., 2012[95]). This indicates that NO-induced vasodilation is mediated through the GC/cGMP/ PKG pathway. Inhibition of NOS by LNAME, was shown to decrease whole pancreatic and, in particular, islet blood flow in normal and diabetic rats (Svensson et al., 1994[124]). Indeed, to accommodate the elevated demand for insulin delivery into the peripheral circulation, islet capillaries expand through dilation but not by angiogenesis (Dai et al., 2013[24]). 

Taken together, emerging evidence has demonstrated that NO in the β-cells, through increasing cGMP and intracellular Ca^2+ ^levels, or via *S*-nitrosilation of glucokinase and syntaxin 4 as well as vasodilation of islet vasculature, increases insulin secretion. 

### Inhibitory effects of NO on insulin secretion

Suppression of mouse islet NOS activity (by L-NAME, L-NMMA, or 7-nitroindazole), is accompanied by an increase in GSIS (Panagiotidis et al., 1995[100]; Akesson et al., 1999[1]; Henningsson et al., 2000[49], 2002[52]; Eckersten and Henningsson, 2012[28]), indicating the negative role of NO in insulin secretion. In addition, GSIS has been shown to be suppressed by different concentrations of NO donors in isolated mouse (Panagiotidis et al., 1995[100]; Akesson and Lundquist, 1999[2]) and rat (Antoine et al., 1996[5]) pancreatic islets. 

**Figure 2:**
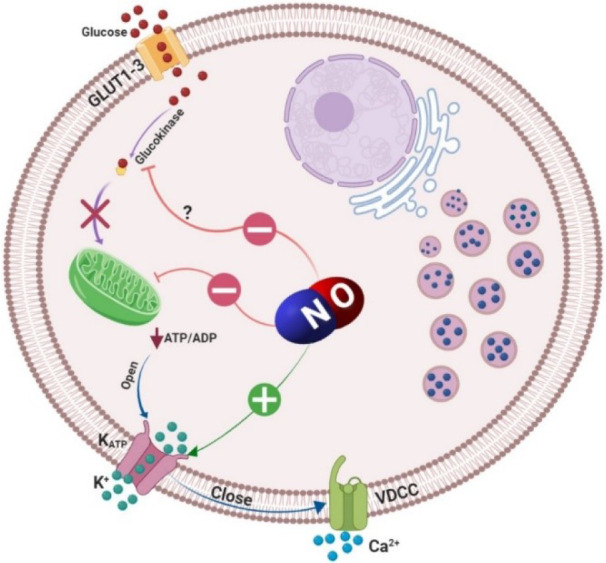
Inhibitory effects of nitric oxide (NO) on insulin secretion. Glucose that enters the β‐cells via glucose transporter type 2 (GLUT-2) is phosphorylated by glucokinase and its metabolism through Krebs cycle, increases cytoplasmic adenosine triphosphate (ATP)/adenosine diphosphate (ADP) ratio. An increased ATP/ADP ratio closes ATP‐sensitive K^+^ channels (K_ATP_ channels) and causes membrane depolarization and the subsequent activation of L‐type voltage‐dependent Ca^2+^ channels (VDCC); elevation of cytosolic Ca^2+^ level leads to insulin vesicles exocytosis. Nitric oxide by formation of ironnitrosyl complexes with FeS-containing enzymes causes reversible inactivation of the mitochondrial enzyme aconitase; NO also reacts with a number of sulfhydryl-containing proteins; functionally essential SH groups in glucose binding site of glucokinase is a target for oxidizing agents. Furthermore, NO increases K^+^ outflow from the β-cells and therefore decreases Ca^2+^ entry through VDCC which inhibits insulin secretion.

Concerning the mechanism, by which NO inhibits insulin secretion, there is evidence showing that NO acts through the formation of S-nitrosothiols (Stamler et al., 1992[121]), as well as changes in transmembrane ionic movements (Figure 2(Fig. 2)) (Antoine et al., 1996[5]). Hydroxylamine was shown to decrease Ca^2+^ entry through voltage-sensitive Ca^2+^ channels and increase K^+ ^outflow from pancreatic islets, an effect which is impaired by glibenclamide (Antoine et al., 1996[5]). Hydroxylamine, however, does not affect Ca^2+ ^outflow and [Ca^2+^]_i_ rises evoked by K^+^-induced depolarization; the enhancing effect of hydroxylamine on K^+^ outflow as well as its lack of effect on the cationic responses to K^+^ stimulation, indicate that the decrease of Ca^2+^ entry can be regarded as the consequence of K^+^ channel activation (Antoine et al., 1996[5]). In addition, NO reacts with a number of sulfhydryl containing proteins (Stamler et al., 1992[121]), and functionally essential SH groups in the glucose-binding site of glucokinase is a target for oxidizing agents (Lenzen et al., 1988[73]). Furthermore, NO by formation of ironnitrosyl complexes with FeS containing enzymes such as aconitase, causes reversible inactivation of the mitochondrial enzyme (Lancaster and Hibbs, 1990[69]). Such mechanisms have been proposed by studies on the inducible form of NO synthase in insulin producing cells (Eizirik and Leijerstam, 1994[29]).

## Controversy about the Role of NO in Insulin Secretion

Much of the confusions about the role of NO in insulin secretion emanates from the use of different and, perhaps, discrepant model system, such as β-cell lines with different qualitative/quantitative secretory reaction patterns compared with normal β-cells, incubation of islets/β-cell lines in high or low glucose, the use of different NOS inhibitors or different types of extracellular/intracellular NO donors, as well as variable enzymatic activities of the different isoforms of NOS expressed in islets. 

On balance, cNOS-derived NO seems to stimulate insulin release while iNOS-derived NO inhibits insulin secretion (Nystrom et al., 2012[95]; Sansbury and Hill, 2014[109]). The conversion of L-arginine to NO and increased insulin secretion in cell-free preparations of HIT-T15 cells appear to be due to the activity of cNOS, as the Ca^2+^ ionophore A23187, which induces insulin secretion (Conaway et al., 1976[19]), also induces NO release from HIT-T15 cells (Schmidt et al., 1992[110]). In addition, insulin secretion is correlated with the intracellular concentration of NADPH (Attie, 2015[7]), Ca^2+^ (Conaway et al., 1976[19]), and calmodulin (Dadi et al., 2014[23]) which all are necessary for cNOS activation. Low concentrations of NO elevate ATP production and cause K_ATP_ channel closure, while high concentrations of NO are associated with a decrease in ATP production, independent of cGMP (Sunouchi et al., 2008[123]). Overproduction of NO by iNOS contributes to islet dysfunction, impairing several critical sites in the β-cell, such as the mitochondrial electron transfer chain, nuclear DNA, tricarboxylic acid cycle aconitase, and transmembrane ion channels (Welsh and Sandler, 1992[132]; Dunger et al., 1996[27]; McDaniel et al., 1996[87]; Eizirik and Pavlovic, 1997[30]). In contrast, silencing of iNOS expression has been demonstrated to exert a protective effect on rat pancreatic islets exposed to proinflammatory cytokines (Bai-Feng et al., 2010[11]). Moreover, NO, when produced by iNOS, induces apoptosis in pancreatic β-cells through a cGMP-mediated pathway (Loweth et al., 1997[75]). Pancreatic β-cells are particularly sensitive to damage by high levels of NO and free radicals because of their low levels of anti-oxidant enzymes such as superoxide dismutase, catalase, and glutathione peroxidase (Wang and Wang, 2017[128]). 

The concentration of glucose, used in studies, may be another reason for the discrepant effects of NO on insulin secretion: at low glucose concentration (7 mM), L-NAME has no effect on insulin secretion in isolated islets from freely fed mice but slightly stimulates insulin secretion in islets from starved animals. At high glucose concentrations (20 mM), L-NAME potentiates insulin secretion in islets from both freely fed and fasted mice (Eckersten and Henningsson, 2012[28]). Regarding the overexpression of iNOS at high glucose concentrations, increased insulin secretion by L-NAME may be due to inhibition of iNOS. Another factor that should be taken into account is the concentrations of NOS inhibitors used in the studies. The effect of L-NAME on insulin secretion seems to be concentration-dependent, as concentrations of 0.1-5.0 mM did not significantly influence basal insulin release in mouse isolated islets while at a high concentration (10 mM), it induced a 3-fold increase in insulin secretion (Panagiotidis et al., 1995[100]).

## Conclusion

Pancreatic β-cells increase expression of iNOS in response to inflammatory stimuli. This produces high cytotoxic NO levels, leading to β-cell damage, dysfunction, and cell death, which may be of relevance in the pathogenesis of both type 1 and type 2 diabetes. Expression of cNOS in β-cells is supported by a large body of evidence. Given the stimulatory effects of the amino acid L-arginine, a precursor of NO, on insulin release, it has been suggested that low cNOS-derived NO levels in β-cell are involved in the regulation of insulin release. However, several studies, in which cNOS activity was manipulated or exogenous NO was applied, have reported both physiological and pathological actions of NO with respect to insulin secretion. Inhibition of NOS decreased, increased, or had no effect on insulin secretion. Similarly, exogenously applied NO had stimulatory or inhibitory effects on insulin secretion. These discrepant data on the role of NO in insulin secretion may be the result of variability of the experimental conditions, e.g., use of various β-cell lines with different qualitative/quantitative secretory patterns, incubation of islets/β-cell lines in high or low glucose, differences in NOS inhibitors or types of extracellular/intracellular NO donors with different pharmacokinetics and pharmacodynamics, the concentration of these agents, as well as enzymatic activities of the different isoforms of NOS. All these features underlie the discrepant results and must be properly considered in future studies of NO in diabetes research.

## Declaration of interest

The authors report no conflict of interest.

## Acknowledgements

This study was supported and approved by the Research Institute for Endocrine Sciences (grant No. 97060), Shahid Beheshti University of Medical Sciences, Tehran, Iran. We thank Professor Hindrik Mulder for constructive feedback and Alexander Hamilton for English editing.

## Authors' contributions

Conceptualization: Sevda Gheibi and Asghar Ghasemi. Writing - original draft: Sevda Gheibi. Writing - review & editing: Sevda Gheibi and Asghar Ghasemi.

## Figures and Tables

**Table 1:**
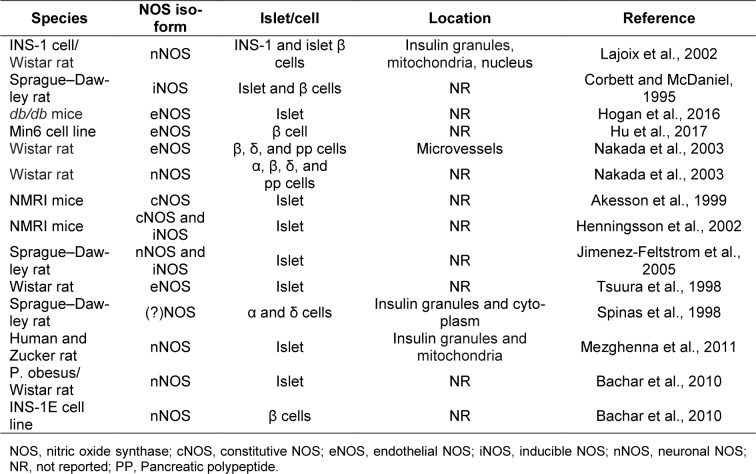
Expression of NOS isoforms in the endocrine pancreas

**Table 2:**
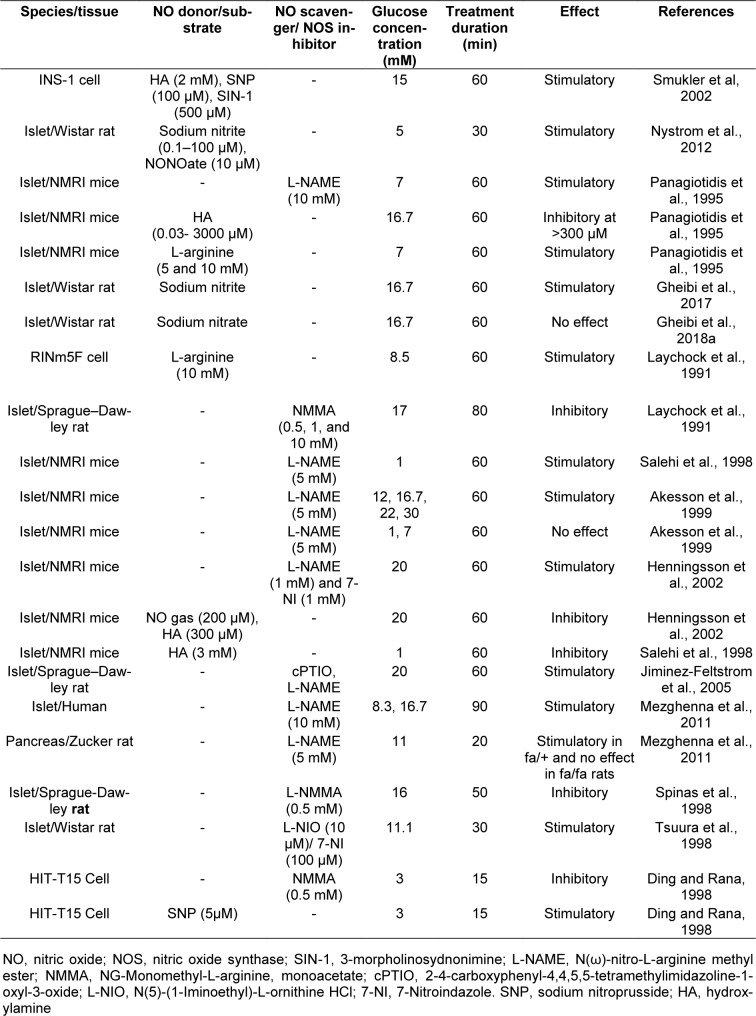
Effects of nitric oxide on insulin secretion
